# Distinct Signalling Pathways of Murine Histamine H_1_- and H_4_-Receptors Expressed at Comparable Levels in HEK293 Cells

**DOI:** 10.1371/journal.pone.0107481

**Published:** 2014-09-22

**Authors:** Silke Beermann, Marcus Vauth, Rabea Hein, Roland Seifert, Detlef Neumann

**Affiliations:** Institute of Pharmacology, Hannover Medical School, Hannover, Germany; Universidade Federal do Rio de Janeiro, Brazil

## Abstract

Histamine (HA) is recognized by its target cells via four G-protein-coupled receptors, referred to as histamine H_1_-receptor (H_1_R), H_2_R, H_3_R, and H_4_R. Both H_1_R and H_4_R exert pro-inflammatory functions. However, their signal transduction pathways have never been analyzed in a directly comparable manner side by side. Moreover, the analysis of pharmacological properties of the murine orthologs, representing the main targets of pre-clinical research, is very important. Therefore, we engineered recombinant HEK293 cells expressing either mouse (m)H_1_R or mH_4_R at similar levels and analyzed HA-induced signalling in these cells. HA induced intracellular calcium mobilization *via* both mH_1_R and mH_4_R, with the mH_1_R being much more effective. Whereas cAMP accumulation was potentiated *via* the mH_1_R, it was reduced *via* the mH_4_R. The regulation of both second messengers *via* the H_4_R, but not the H_1_R, was sensitive to pertussis toxin (PTX). The mitogen-activated protein kinases (MAPKs) ERK 1/2 were massively activated downstream of both receptors and demonstrated a functional involvement in HA-induced EGR-1 gene expression. The p38 MAPK was moderately activated *via* both receptors as well, but was functionally involved in HA-induced EGR-1 gene expression only in H_4_R-expressing cells. Surprisingly, in this system p38 MAPK activity reduced the HA-induced gene expression. In summary, using this system which allows a direct comparison of mH_1_R- and mH_4_R-induced signalling, qualitative and quantitative differences on the levels of second messenger generation and also in terms of p38 MAPK function became evident.

## Introduction

HA, a biogenic amine, is an important mediator of many physiological and pathological processes such as gastric acid secretion, neurotransmission, cell differentiation, immunomodulation, allergic reactions, peptic ulcer, and tumor progression [1]. Two groups of cells are able to produce HA. The first group comprises mast cells, basophils, histaminergic neurones and ECL-cells of the gastric wall, which are able to store HA in intracellular granules. Specific stimuli lead to degranulation of these cells and to a massive release of HA [2], [3]. The second group of HA-producing cells consists of neutrophils, lymphocytes, macrophages and others. These cells secrete HA immediately following its production, which is regulated by specific stimuli *via* expression of the histamine-generating enzyme L-histidine decarboxylase [4], [5].

Cellular effects of HA are mediated *via* four G-protein coupled receptors (GPCRs), H_1_R - H_4_R, which are expressed on varying cell types. H_x_Rs differ from each other in their preferred coupling G-protein and, thus, activate different signal transduction pathways. Accordingly, H_X_Rs carry out different functions in the body [6]–[9].

The H_1_R is expressed ubiquitously, including several immune cells such as B- and T-cells, monocytes, macrophages and dendritic cells [10], [11]. Upon ligand binding the H_1_R couples to G_q_-proteins and activates phospholipase C (PLC) [12], followed by the generation of inositol-1,4,5-trisphosphate (IP_3_) and diacylglycerol (DAG) of which IP_3_ induces an increase in intracellular Ca^2+^ concentrations ([Ca^2+^]_i_) [10], [13]–[15]. Moreover, the activated H_1_R potentiates forskolin-induced production of the second messenger cAMP [16]. This increase is either mediated *via* activation of specific adenylylcyclase (AC) isoforms by Ca^2+^-activated calmodulin [17] or *via* a Ca^2+^-independent direct activation of ACs [18]. Further downstream, H_1_R mediated signalling involves also MAPKs [19], [20].

The H_4_R is mainly expressed on immune cells [21]–[25], thus can be detected in virtually all organs [26]. The H_4_R plays a role in inflammatory- and immunoreactions e.g. by inducing chemotaxis in mast cells, T-cells, eosinophils, macrophages, and dendritic cells [22]–[24], [27], [28]. Upon ligand binding the H_4_R activates pertussis toxin-sensitive G_i_-proteins [29], [30] activating PLC which catalyses the hydrolysis of phosphatidylinositol-4,5-bisphosphate (PIP_2_) to IP_3_ which causes an increase in [Ca^2+^]_i_ [23], [31]. Furthermore, the activated G_i_-protein directly inhibits ACs and, thereby, decreases *e.g.* forskolin-induced cAMP concentrations [32], [33]. Moreover, similar to the H_1_R stimulation, H_4_R activates MAPK signalling pathways [30], [34].

H_4_R antagonists such as JNJ 7777120 [21], [35] have already been successfully used pre-clinically in animal models. Application of H_4_R antagonists as well as genetic deletion of the H_4_R in a mouse asthma model improved asthmatic symptoms. Our own previous studies in murine asthma demonstrated that the H_1_R antagonist mepyramine affects the ameliorating effect of the H_4_R antagonist JNJ7777120 [35]. Thus, understanding the signalling pathways activated by the murine (m)H_1_R and mH_4_R is of high interest. However, so far H_X_R-signalling was mainly analysed for the human orthologs while comprehensive information on the murine receptors is missing. In contrast, H_X_R ligands are analysed pre-clinically mainly in mouse models. Therefore, it is important to compare biochemical and pharmacological parameters in both systems, human and mouse, to estimate if effects that are observed in mice can be translated to humans. In order to analyse mH_1_R and mH_4_R signalling, we generated HEK293 cells, which are devoid of endogenous histamine receptors, stably expressing comparable levels of either recombinant mH_1_R or mH_4_R. These cells were incubated with HA, the H_1_R- and H_4_R-specific antagonists mepyramine and JNJ7777120, and specific inhibitors of MAPKs. The resulting signalling activities were analysed with respect to mobilization of intracellular caclcium, intracellular cAMP accumulation, activation of MAPKs and EGR-1 gene expression.

## Materials and Methods

### Materials

If not stated otherwise, all chemicals were obtained from Sigma-Aldrich (Taufkirchen,, Germany). PCR primers were purchased from Eurofins MWG Operon (Ebersberg, Germany). The MAPK inhibitors SB 203580 and PD 98059 were purchased from Tocris (Bristol, United Kingdom), and pertussis-toxin from List Biological Laboratories (Campbell, CA, USA). The H_4_R-selective antagonist JNJ7777120 (1-[(5-chloro-1H-indol-2-yl) carbonyl]-4-methylperazine) was kindly provided by Dr. Armin Buschauer (University of Regensburg, Germany).

### HEK293 cells and generation of stably H_X_R-expressing clones

HEK293 cells (LGC Standards (ATCC), Wesel, Germany) were maintained in DMEM medium (PAA Laboratories, Pasching, Austria) supplemented with 10% [v/v] FCS (Lonza, Basel, Switzerland), penicillin (100 units/ml)/streptomycin (100 µg/ml) and 2 mM L-glutamine at 37°C in a 7% [v/v] CO_2_ environment. Confluent cell layers were split twice a week.

Cells at 60–80% confluency were transfected with pcDNA3 plasmids containing the coding sequences for either myc-tagged mH_1_R or Flag-tagged mH_4_R, or the empty vectors using Fugene HD (Roche, Mannheim, Germany) according to the manufacturer's protocol. Cells with stable integration of vectors or plasmids were selected with G418 (empty vector, mH_1_R) or Zeocin (empty vector, mH_4_R) (both Invivogen, San Diego, CA, USA). Clones were generated by limiting dilution technique and receptor expression was checked by flow cytometry (Miltenyi Biotech, Bergisch Gladbach, Germany) and Western blot using anti-Flag and anti-myc (Roche,Penzberg, Germany) reagents.

### Detection of intracellular Ca^2+^ concentration

HEK293 mH_X_R cells were incubated at a density of 1×10^7^ cells/ml in Krebs-Hepes buffer (120 mM NaCl, 20 mM Hepes, 4.7 mM KCl, 1.2 mM KH_2_PO_4_, 1.2 mM MgSO_4_, 1.25 mM CaCl_2_, 10 mM glucose, pH 7,4) containing 10 µM Fura-2-AM (Tocris, Bristol, United Kingdom) and 0.2% [m/v] pluronic F127 for 40 min under rotation at room temperature in the dark. Then, cells were diluted 1∶10 with Krebs-Hepes buffer and incubated for an additional 20 min at the same conditions. Cells were sedimented by centrifugation and resuspended at 2×10^7^ cells/ml in fresh Krebs-Hepes buffer. Labelled cells were seeded in a black 96 well plate at 50 µl/well and incubated with increasing concentrations of mepyramine or JNJ7777120, or with Krebs-Hepes buffer for a few minutes. Fluorescence measurement was performed in a Synergy 4 reader (Biotek, Bad Friedrichshall, Germany) with high frequently alternating excitation wavelengths of 340 and 380 nm and an emission wavelength of 508 nm. After 2 min of detection of the baseline, HA at the concentration indicated was added. In antagonists studies, the HA solutions were supplemented with the respective antagonists in order to keep antagonists concentration constant. The effect of HA stimulation on [Ca^2+^]_i_ was measured over a period of 3 min. Then, Triton X-100 at a final concentration of 0.5% (w/v) was added and the maximal signal (F_max_) was detected over a period of 2 min. Finally, EGTA was added at a final concentration of 12 mM and the minimal signal (F_min_) was detected for additional 2 min. The increase in [Ca^2+^]_i_ was calculated from these data using the following equitation:
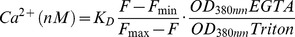



### Quantification of intracellular cAMP accumulation using HPLC/MS/MS

HEK293 mH_X_R cells were seeded in 6-well plates at 1×10^6^ cells/well and cultured for 24 h. Cells were stimulated for 10 min at 37°C with 100 µM forskolin and 100 µM HA in the presence or absence of mepyramine and/or JNJ7777120 at the concentrations indicated. The medium was removed and 300 µl of extraction reagent (AcN/MeOH/H_2_O (2∶2∶1)) containing 25 ng/ml tenofovir (internal standard) was added to the wells. The extracts were incubated at 98°C for 20 min and aggregated protein was collected by centrifugation for 10 min at 17000 g. Supernatant fluids were transferred into a new tube and evaporated at 40°C under nitrogen flow until complete dryness and residual material was solved in 150 µl H_2_O. Samples were diluted 1∶100 in H_2_O containing 50 ng/ml tenofovir and analyzed on an API 5500 (AB SCIEX, Framingham, MA, USA) mass spectrometer after HPLC-separation using a Zorbax Eclipse column XDB-C18 1.8 µ 50×4,6 (Agilent Technologies, Santa Clara, CA). cAMP concentrations of samples were calculated according to standards containing defined cAMP concentrations. The protein pellets were dried at RT and solved in 800 µl 0.1 M NaOH at 95°C for 30–60 min. Protein concentrations were quantified using BCA-assay (Thermo Fischer Scientific, Waltham, MA, USA). cAMP concentrations were calculated in relation to the protein concentration (pmol cAMP/mg protein).

### MAPK array

HEK293 mH_X_R cells were seeded in 6-well plates at 1×10^6^ cells/well and cultured for 24 h. After incubation with or without 10 µM HA for 5 min, cells were analyzed using the MAPK array according to the manufacturer's protocol (Human Phospho-MAPK-Array; R&D System, Minneapolis, MN, USA [36]). Protein concentrations of cellular lysates were determined by Bradford protein assay (BioRad Laboratories) and 300 µg protein were subjected to each membrane. For quantitative analysis, the pixel density of every individual spot was determined and intensities of the spots without HA-stimulation were subtracted from the intensities of the corresponding spots with HA-stimulation.

### RNA extraction and real time PCR

HEK293 mH_X_R cells were seeded in 6-well plates at 1×10^6^ cells/well and cultured for 24 h. After pre-incubation with or without MAPK inhibitors followed by stimulation with or without 10 µM HA (as indicated) for 4 h, the medium was removed and the cells were washed twice with PBS. Total RNA was extracted using the NucleoSpin RNA II kit (Macherey-Nagel, Düren, Germany) according to the manufacturer's protocol. Two µg RNA were reverse transcribed into cDNA using oligo dT primers (Fermentas, Rockford, IL, USA) and RevertAid Reverse Transcriptase (Fermentas).

Real time PCR was performed by the TaqMan method. Buffers and TaqMan probes were purchased from Applied Biosystems (Darmstadt, Germany) and the assay was performed according to the manufacture's protocol. For standardisation the house-keeping genes β-actin and GUS-β were amplified and gene expression was quantified using the ΔΔCt method [37].

### Statistical analysis

If not stated otherwise, statistical analyses were performed by calculating means ± SD of at least three independent determinations. Analysis of significance was performed using Student's t-test or one-way ANOVA with Bonferroni post-test for linear parameters (GraphPad Prism 5). p-Values of ≤0.05 (*), ≤0.01 (**) and ≤0.005 (***) were considered significant.

## Results

### Histamine increases intracellular Ca^2+^ concentrations via mH_1_R and H_4_R with different potencies and efficacies

**Figure 1:**
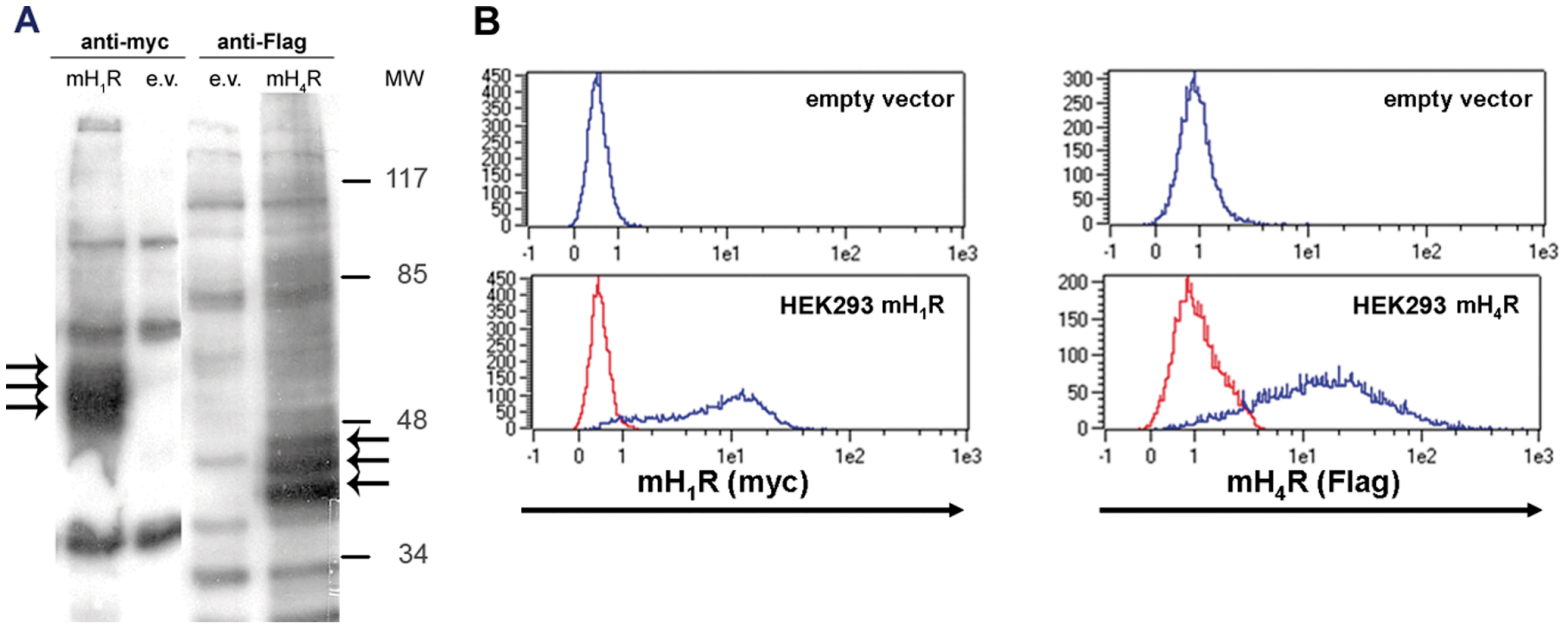
Successful generation of HEK293 cells stably expressing mH_1_R and mH_4_R. HEK293 cells were transfected with plasmids encoding the myc-tagged mH_1_R, the Flag-tagged mH_4_R, or the empty vectors (e.v.) as indicated and clones thereof were generated by limiting dilution technique. Stable expression of the transgenes was anlayzed by Western blot (A) and flow cytometry (B) using myc- and Flag-specific reagents as indicated. Presented are data from a single clone of each cell line, which has been chosen for further analyses. Arrows in (A) indicate the recombinantly expressed proteins.

Clones of HEK293 cells stably expressing epitop-tagged fusion proteins of either mH_1_R or mH_4_R were generated. A myc-tag was added N-terminally at the mH_1_R, and the mH_4_R was flanked by a Flag-tag. The presence of the receptor proteins encoded by the exogenous genes was analyzed by Western blot (Figure 1A) and flow cytometry (Figure 1B). The appearance of several species with differing apparent molecular weights (Figure 1A) is due to differing glycosylation of the receptor proteins [38], [39]. For functional analyses, individual clones of both cell lines demonstrating comparable quantities of receptor expression were chosen. This quantification relayed on flow cytometry since radioligand-binding analysis is not feasible for the mH_4_R due to the lack of a suitable radio-labelled ligand [8]. The chosen clones are referred to as HEK293 mH_1_R and HEK 293 mH_4_R.

**Figure 2:**
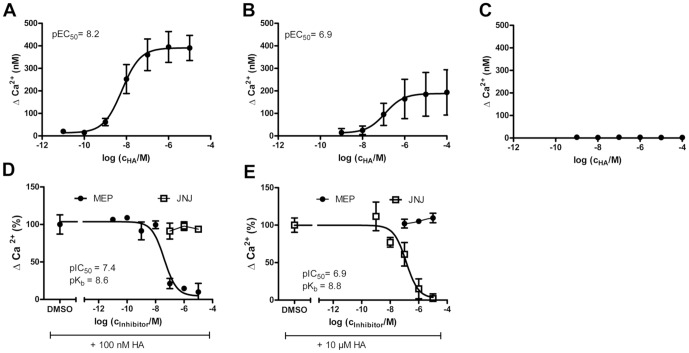
Mobilization of [Ca^2+^]_i_ by histamine is mediated by H_1_R and H_4_R. HEK293 mH_1_R cells (A+D), HEK293 mH_4_R cells (B+E), and empty vector-transfected HEK293 cells (C) were labelled with Fura 2-AM and stimulated with different concentrations of histamine (HA) (A–C) or with a constant HA concentration in combination with DMSO or varying concentrations of mepyramine (MEP) or JNJ7777120 (JNJ) (D+E). Changes in [Ca^2+^]_i_ were determined by fluorescence measurements at an emission wavelength of 508 nm and excitation wavelengths of 340 nm and 380 nm. Data shown are means ± SD (n = 2–3, each consisting of 2 replicates).

In HEK293 mH_1_R and HEK293 mH_4_R cells HA increased [Ca^2+^]_i_ concentration-dependently (Figure 2A,B). While in HEK293 mH_1_R a maximal Δ[Ca^2+^]_i_ of about 400 nM (Figure 2A) was reached, the maximum Δ[Ca^2+^]_i_ of HEK293 mH_4_R cells was only 200 nM (Figure 2B). Moreover, HEK 293 mH_1_R cells displayed a substantially higher potency for HA than HEK293 H_4_R cells, as reflected by a pEC_50_ of 8.2 and of 6.9, respectively. In contrast, in empty vector-transfected HEK293 cells HA did not increase [Ca^2+^]_i_ (Figure 2C).

The selective antagonists mepyramine (H_1_R) and JNJ7777120 (H_4_R) were used to document the receptor specificity of HA-induced increased [Ca^2+^]_i_. In HEK293 mH_1_R cells the elevated [Ca^2+^]_i_ was reduced concentration-dependently by mepyramine with a pIC_50_ of 7.4 and a pK_B_ of 8.6, while JNJ7777120 was without effect (Figure 2D). JNJ7777120, but not mepyramine, inhibited the HA-induced increase in [Ca^2+^]_i_ in HEK293 mH_4_R cells displaying a pEC_50_ of 6.9 and a pK_B_ of 8.8 (Figure 2E). Thus, the HA-induced increase of [Ca^2+^]_i_ in the transfected HEK 293 cells is mediated indeed by the respective recombinant receptors and not by an unspecific activity of HA.

### Intracellular cAMP concentrations are enhanced via mH_1_R and decreased via mH_4_R

**Figure 3:**
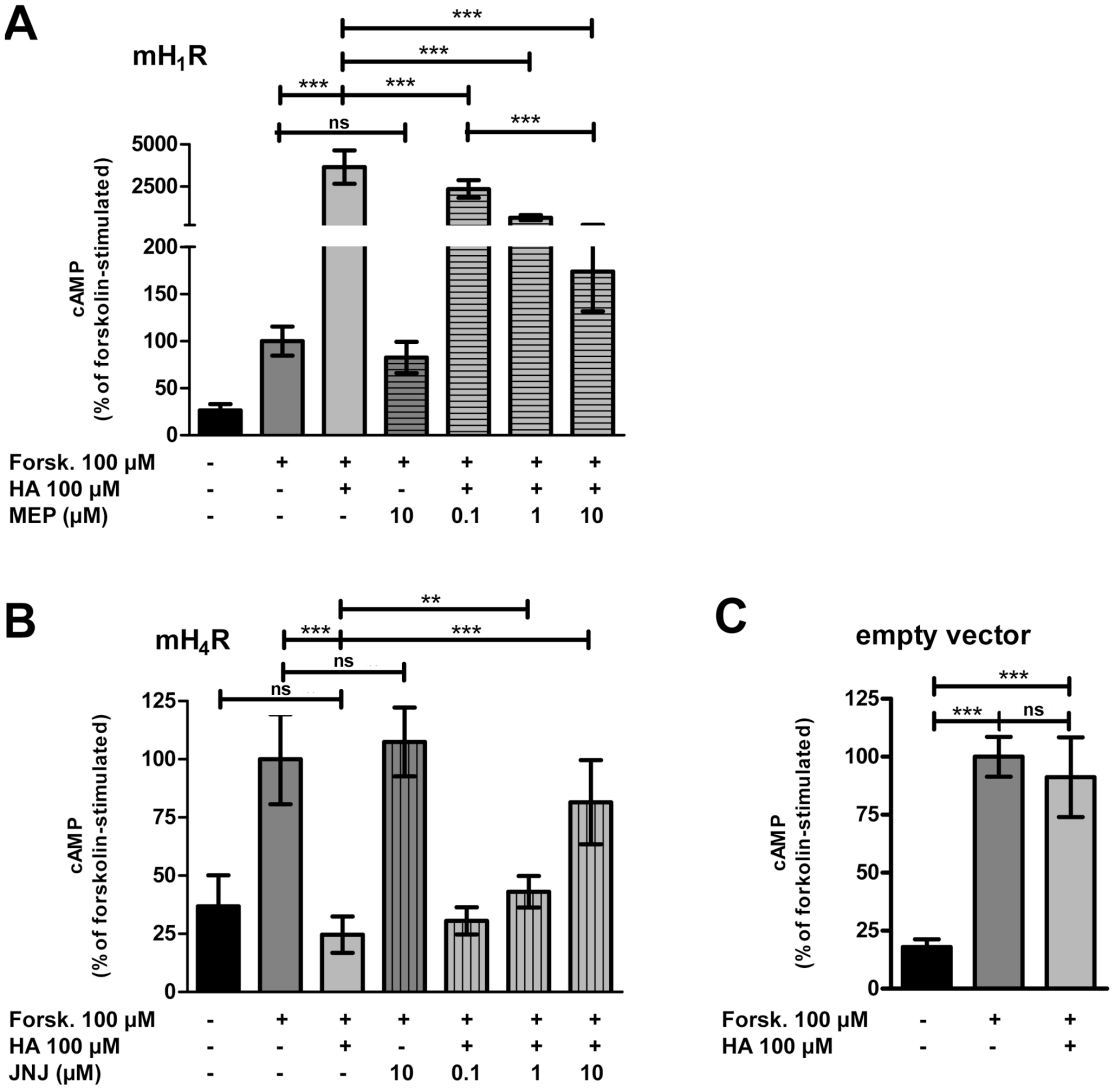
Intracellular cAMP concentrations are enhanced by histamine *via* the H_1_R and reduced by HA *via* the H_4_R. HEK293 mH_1_R cells (A), HEK293 mH_4_R cells (B), and empty vector-transfected HEK293 cells (C) cells were stimulated with forskolin (Forsk) and histamine (HA) in the absence or presence of mepyramine (MEP) or JNJ7777120 (JNJ) for 10 min. Intracellular cAMP was extracted from the cells and the concentration was determined using HPLC/MS/MS and calculated in relation to the protein concentration. The cAMP-concentration after stimulation with forskolin (H_1_R: 186±57 pmol/mg protein; H_4_R: 309±111 pmol/mg protein) was set to 100 % and other values were calculated on this base. Data shown are means ± SD (n = 2–5, each consisting of 3 replicates; *: p≤0.05; **: p≤0.01; ***: p≤0.005; ns: not significant).

cAMP is generated by AC, which can be activated directly by forskolin [9], [40]. In comparison to untreated controls, stimulation with forskolin elevated the cAMP-concentration in HEK 293 mH_1_R cells, HEK 293 mH_4_R cells, and empty vector-transfected cells, which were defined as 100 % for relative quantification (Figure 3A,B). Additional stimulation with 100 µM HA enhanced cAMP accumulation in HEK293 mH_1_R cells to about 3500 %. This enhancement was reduced by mepyramine in a concentration dependent manner (Figure 3A). In HEK293 mH_4_R cells, HA reduced the forskolin-induced cAMP-concentration to basal levels. This effect was concentration-dependently diminished by JNJ7777120 (Figure 3B). The antagonists mepyramine and JNJ7777120 both at concentrations up to 10 µM were selective for their respective receptors since the HA effects were neither affected by mepyramine in HEK293 mH_4_R cells nor by JNJ777120 in HEK293 mH_1_R cells (data not shown). Finally, in empty vector-transfected cells, HA did not affect forskolin-induced cAMP generation (Figure 3C) and stimulation with HA alone enhanced cAMP accumulation in HEK293 mH_1_R cells while it had no effect in HEK293 mH_4_R cells and empty vector –transfected cells (data not shown).

### Pertussis toxin inhibits mH_4_R- but not mH_1_R-mediated effects

Experiments with pertussis toxin (PTX), which selectively inhibits G_i_-protein activation by GPCRs [41], [42], were performed. HEK293 mH_1_R and HEK293 mH_4_R cells were incubated with or without PTX for 18 h and then the [Ca^2+^]_i_ and the forskolin-induced cAMP concentrations were evaluated after stimulation with HA.

**Figure 4:**
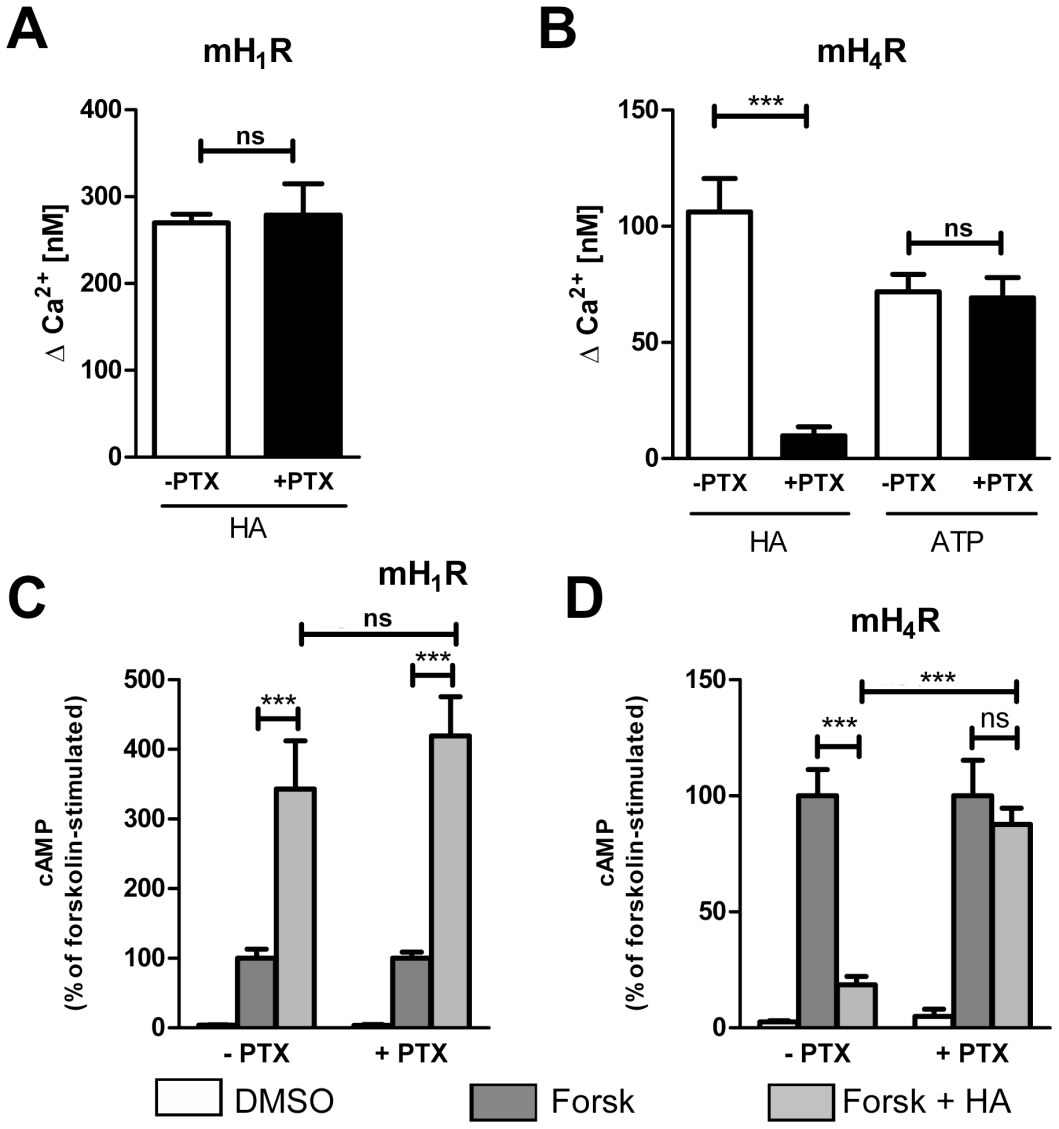
Pertussis toxin selectively blocks signaling of the mH_4_R. HEK293 mH_1_R cells (A+C) and HEK293 mH_4_R cells (B+D) were incubated in the presence or absence of 50 ng/ml pertussistoxin (PTX) for 18 h. Changes in [Ca^2+^]_i_ were determined as described in Figure 2 after stimulation with 100 µM histamine (HA) or 10 µM ATP as indicated on the x-axis (A+B). Intracellular cAMP concentrations were analyzed as described in Figure 3 after stimulation for 10 min with 100 µM forskolin (Forsk) and histamine (HA) (C+D). Forskolin-induced cAMP concentrations were 2385±160 pmol/mg protein in HEK293 H_1_R and 2164±436 pmol/mg protein in HEK 293 H_4_R cells, respectively. Data shown are means ± SD (n = 2, each consisting of 2–3 replicates; *: p≤0.05; **: p≤0.01; ***: p≤0.005; ns: not significant).

PTX did not affect the HA-induced increase in [Ca^2+^]_i_ (∼280 nM) in HEK293 mH_1_R cells (Figure 4A). In contrast, stimulation of untreated HEK293 mH_4_R cells with HA enhanced [Ca^2+^]_i_ to about 100 nM, while such increase was absent in PTX pre-treated HEK293 mH_4_R cells (Figure 4B). The remaining Δ[Ca^2+^]_i_ of about 10 nM was consistent with the concentration in unstimulated cells. PTX treatment did not generally inhibit the ability of HEK293 mH_4_R cells to mobilize Ca^2+^ ions, since stimulation with ATP increased [Ca^2+^]_i_ independently of pre-incubation with PTX (Figure 4B).

In HEK293 mH_1_R and HEK293 mH_4_R cells forskolin induced a cAMP increase independently of PTX treatment (Figure 4C,D). In HEK293 mH_1_R cells HA enhanced forskolin-induced cAMP concentrations up to 400 % in the absence as well as in the presence of PTX treatment (Figure 4C). HEK293 mH_4_R cells showed an 80 % reduction of forskolin-induced cAMP concentrations after stimulation with HA which was absent upon pre-incubation with PTX (Figure 4D). Thus, intracellular Ca^2+^ mobilization and cAMP generation is affected by HA *via* both mH_1_R and mH_4_R, but with differing quantities (Ca^2+^) and qualities (cAMP). Moreover, only the mH_4_R, but not the mH_1_R acts in a PTX-sensitive manner.

### HA-induced phosphorylation of MAPKs differs between mH_1_R- and mH_4_R-expressing HEK293 cells

In order to analyse signalling events that occur more distally than the enhancement of intracellular Ca^2+^ and cAMP, the phosphorylation of several MAPKs in lysates of 5 min HA-stimulated HEK293 mH_1_R and HEK293 mH_4_R cells was assessed.

**Figure 5:**
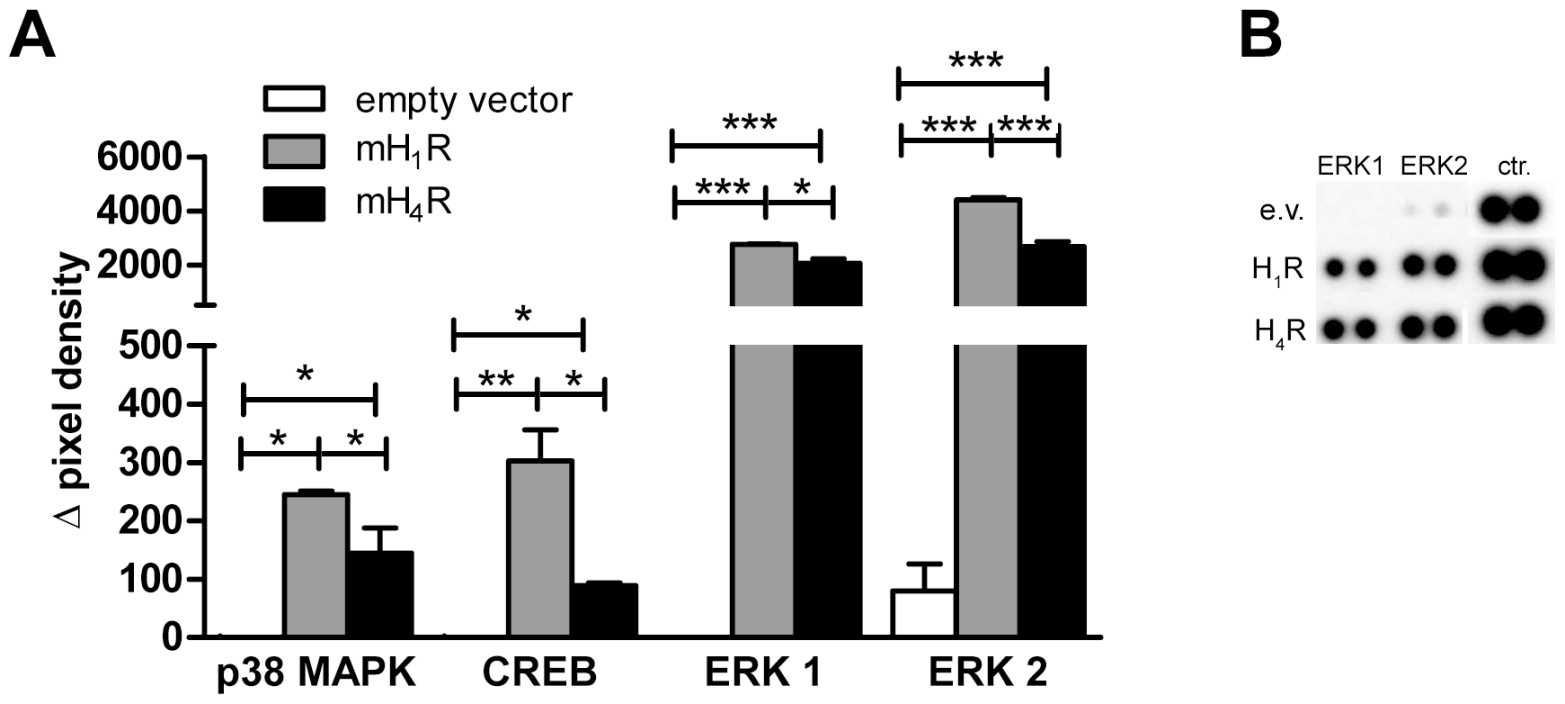
Histamine induces phosphorylation of MAP-kinases *via* H_1_R and H_4_R. HEK293 empty vector cells, HEK293 mH_1_R cells, and HEK293 mH_4_R cells were incubated with or without 10 µM HA for 5 min and lysed afterwards. Phosphorylation of different serine/threonine kinases in these lysates were detected by MAPK array. Phosphorylation intensity was quantified by analysis of the pixel density of every single spot (A). Shown are the differences of the densities of corresponding spots obtained using lysates of cells with and without HA stimulation. Data shown are means ± SD (n = 2, each measured in 2 replicates; *: p≤0.05; **: p≤0.01; ***: p≤0.005). In B, exemplarily the spots of ERK1 and ERK2 after histamine induction in the cells as indicated on the left, and quantification control spots (ctr.) are demonstrated.

In HEK293 mH_1_R as well as in HEK293 mH_4_R cells a similar strong HA-induced phosphorylation of ERK 1 and ERK 2 was detected. p38α and CREB were only moderately phosphorylated *via* activation of mH_1_R and mH_4_R, the H_1_R demonstrating a stronger activity-inducing potential on p38α and CREB phosphorylation as compared to the H_4_R (Figure 5). Thus, signalling emerging at the mH_1_R and the mH_4_R differs basically at the activation of p38 MAPK and the transcription factor CREB.

### HA induced EGR-1 gene expression in mH_1_R- and mH_4_R-transfected cells

As an example for MAPK-regulated genes, the expression of the EGR-1 gene was quantified in HEK293 mH_1_R and HEK293 mH_4_R cells after 4 h stimulation with HA. Specific kinases involved were analysed by use of inhibitors selective for the MAPKs p38 and ERK, and for CREB.

**Figure 6:**
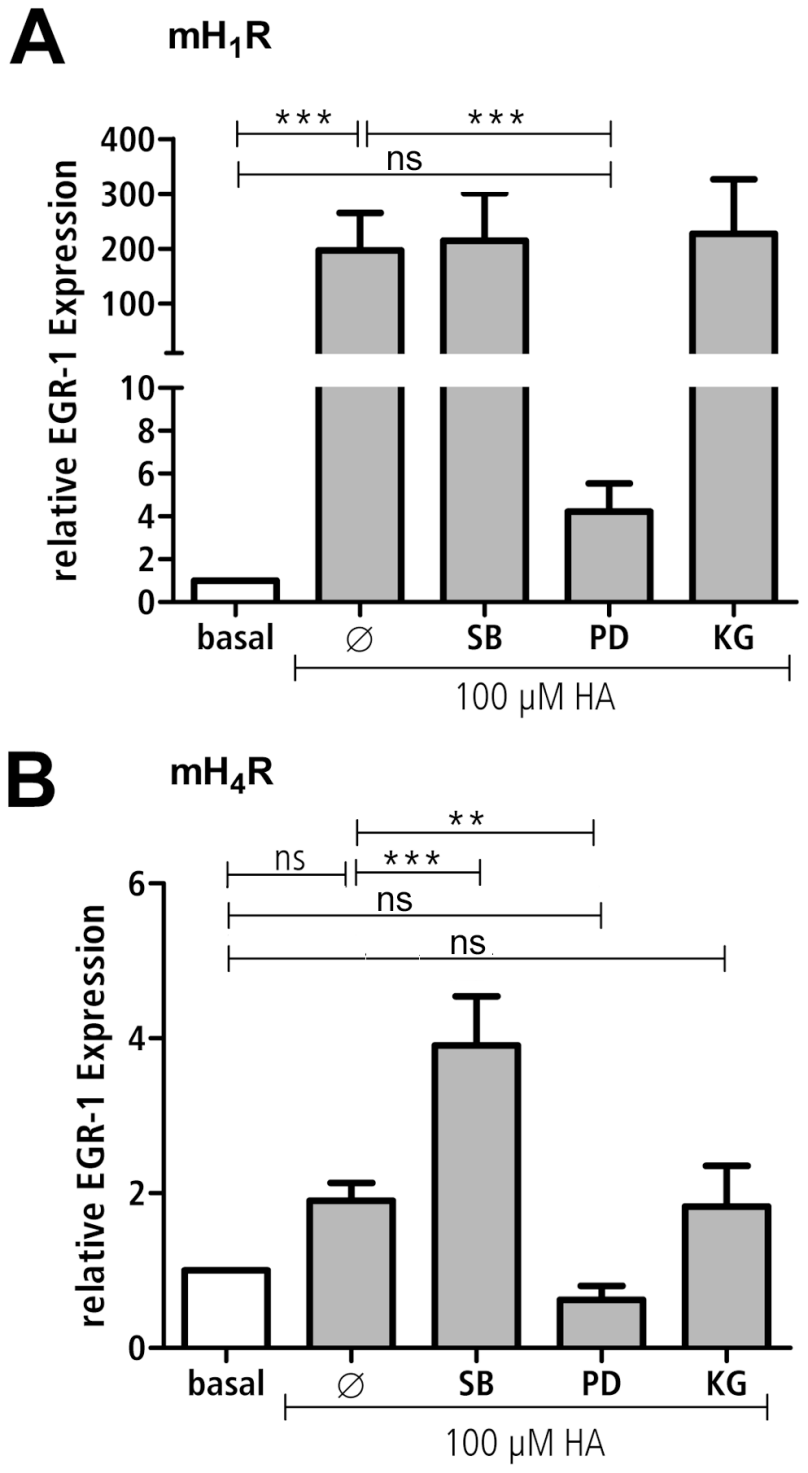
Histamine regulates EGR-1 mRNA expression. HEK293 mH_1_R cells (A) and HEK293 mH_4_R cells (B) were incubated without any inhibitor (Ø) or with 50 µM PD 980598 (PD) for 1 h, with 2 µM SB 203580 (SB) for 30 min, or 25 µM KG-501 (KG) for 20 min and then incubated with or without histamine for 4 h. Total RNA was extracted from the cells and reverse transcribed into cDNA. Expression of EGR-1 was determined by real time PCR using TagMan probes. Data shown are means ± SD of the relative EGR-1 expression in relation to unstimulated cells (basal) (n = 3, each consisting of 2 replicates; **: p≤0.01; ***: p≤0.005; ns: not significant).

EGR-1 expression was enhanced 200-fold in HEK293 mH_1_R cells by stimulation with HA. This HA-induced EGR-1 expression was unaffected by the p38 inhibitor SB 203580 [43] and the CREB inhibitor KG-501 [44]. In contrast, the MEK inhibitor PD 98059, used to assess the involvement of the ERK pathway [43], caused a significant reduction nearly to the level of unstimulated cells (Figure 6A). In HEK293 mH_4_R cells, EGR-1 expression was roughly doubled by stimulation with HA. This statistically not significant effect was unaffected by KG-501 but reduced to the basal level using PD 98059. Interestingly, a significant 4-fold increase of EGR-1 expression was observed upon incubation of HEK293 mH_4_R with HA plus SB 203585 (Figure 6B). The treatment of both HEK293 cell lines with the MAPK inhibitors without HA stimulation did not affect EGR-1 expression, with the exception of HEK293 H_4_R cells incubated with SB 203585, which slightly enhanced EGR-1 expression (data not shown). Thus, HA-induced expression of EGR-1 mediated *via* mH_1_R and mH_4_R involves mainly ERK signalling. In addition, the p38 MAPK pathway seems to reduce mH_4_R-mediated induction of EGR-1 expression.

## Discussion

In the present study we analyzed the signalling pathways of mH_1_R and mH_4_R. A model of stably transfected HEK293 cells expressing either mH_1_R or mH_4_R at comparable levels as assessed by FACS analysis was generated and validated. To the best of our knowledge, signal transduction of the mH_1_R and mH_4_R has never been studied in parallel in a well-controlled expression system before. However, similar expression levels and identical cellular backgrounds are crucial for a direct comparison because the stoichiometry between receptors, G-proteins, and downstream effectors may have a large impact on the signals measured [45]. An advantage of HEK293 cells is their unresponsiveness to HA stimulation because they do not endogenously express functionally active H_x_Rs. In HEK293 cells stably expressing exogenous H_x_Rs, HA-induced signalling was analysed with respect to [Ca^2+^]_i_, intracellular cAMP concentration, activation of MAPKs and EGR-1 gene expression.

Many H_x_R agonists and antagonists are used in different disease models in the mouse. Therefore, it is important to compare pharmacological parameters like affinity and effectivity in both systems, human and mouse, to estimate if effects that are observed in mouse models can be translated to humans. The mouse and human histamine receptor orthologs have a rather low homology (*i.e.* 67 % for H_4_R [46]), and JNJ7777120, which is a partial inverse agonist at the recombinant human H_4_R expressed in an insect cell system, shows partial agonistic effects at the insect cell-expressed recombinant mH_4_R [47], [48]. However, so far no further evidence of an agonistic function of JNJ7777120 in other experimental cellular systems has been observed, specifically in eosinophils, the so far only human cell type with confirmed functional expression of the H_4_R [49]. The concept of functional selectivity, according to which the potency and efficacy of a given ligand depends on the specific signalling pathway studied, may provide a mechanistic explanations for our data [8], [9].

### Regulation of intracellular Ca^2+^ and cAMP concentrations via mH_1_R and mH_4_R

Proximal signalling of mH_1_R and mH_4_R was analyzed by measurements of [Ca^2+^]_i_ and intracellular cAMP concentration. The human H_1_R has been demonstrated to mediate the HA signal to increase [Ca^2+^]_i_ and intracellular cAMP concentration [13], [18], [50]. Stimulation of the human and the murine H_4_R increased [Ca^2+^]_i_ as well [23], [30], [33], while it decreased intracellular cAMP concentrations [23], [32], [51]. Our results on [Ca^2+^]_i_ and the intracellular cAMP concentration regulated by the recombinant mH_1_R and mH_4_R fit well to the above-mentioned literature data. Moreover, we also provide evidence that the mH_4_R expressed in HEK293 cells couples to G_i_-proteins as does the native H_4_R in murine mast cells [23], and that the mH_4_R-generated signal towards regulation of both [Ca^2+^]_i_ and cAMP accumulation is mediated *via* G_i_-proteins. Coupling of the recombinant mH_1_R to G_q_-proteins is highly likely due to the PTX-insensitivity of rises in [Ca^2+^]_i_, excluding the possibility of G_i_-coupling. Because mH_1_R and mH_4_R were expressed at comparable levels, we can also hypothesize that activation of Ca^2+^ signalling via G_q_-proteins is more efficient than via G_i_-proteins. Thus, the mH_1_R may induce a more robust cellular response than the mH_4_R which may act in a more subtle way. In support of this concept, the hH_4_R induces only a small pro-inflammatory response in human eosinophils [52].

### Activation of MAPK signalling pathways via mH_1_R and mH_4_R

Naor et al. [53] reported that different G-proteins can interact in distinct manners with MAPK signalling pathways and, therefore, activate different MAPK cascades. For some GPCRs including the H_1_R- and the H_4_R it has been shown that, in addition to G-protein activation, they activate MAPK signalling pathways independently of G-proteins by direct coupling to β-arrestins [54]–[56]. However, while G-protein dependent phosphorylation appears fast, within minutes after stimulation, β-arrestin-mediated MAPK activation is much slower [57]. Since we analyzed MAPK phosphorylation after 5 min of stimulation, it is very unlikely that β-arrestin signalling is responsible for our observations, thus the effects on MAPK phosphorylation described in this study are rather G-protein mediated. A formal experimental proof, however, is still to be provided.

We found the MAPKs ERK 1/2, and p38, and the transcription factor CREB to be activated in response to HA in both mH_1_R- and mH_4_R-expressing HEK293 cells. The H_1_R has already been described to activate ERK 1/2 and p38 most probably *via* PLC, [Ca^2+^]_i_, protein kinase C and Ras [58]. CREB, the ‘cAMP responsive element binding protein’, is activated by enhanced intracellular cAMP concentration followed by PKA activation [59], [60]. Since in mH_1_R-transfected HEK293 cells HA induced a strong increase in cAMP, CREB phosphorylation can be regarded a direct consequence of this. In HEK293 mH_4_R cells in contrast, histamine stimulation does not enhance cAMP accumulation, but nevertheless, albeit at a very weak level, it activates CREB. This weak cAMP-independent CREB activation may be induced by e.g. pathways emerging from enhanced [Ca^2+^]_i_ mobilization [61], [62].

Phosphorylation of ERK 1/2 has also been detected already in human H_4_R-transfected HEK293 cells [30] and in murine bone marrow-derived mast cells [34]. However, activation of p38 *via* the H_4_R has not been described before and is, thus, a novel finding of this study. In mH_4_R-transfected cells, HA does not induce, but rather reduces cAMP generation. Thus, to detect the phosphorylation of CREB was unexpeceted as well. However, CREB has been found to be activated also by other signalling molecules, such as MAPKAPK-2, a substrate of p38 [58], [63], [64], MSK-1, a substrate of p38 and ERK 1/2 [65], and CaM kinases, which are activated by increased [Ca^2+^]_i_ [62]. Since we found an activation of p38 and ERK 1/2 and enhanced [Ca^2+^]_i_ in mH_4_R-transfected cells upon HA-stimulation, in our system CREB is phosphorylated probably *via* one of these alternative pathways.

### MAPK-mediated gene expression induced by mH_1_R and mH_4_R

The activation of MAPK-signalling pathways finally results in regulation of gene expression. The transcription factor EGR-1 plays a decisive role in inflammatory reactions by the induction of the expression of e.g. IL-6, IL-8, CCL-2, TNF-α and MCP-1 [66]–[68]. In mH_1_R-transfected HEK293 cells, HA induces a strong increase in EGR-1 expression, which is dependent on ERK signalling. This result fits to the data of Guha et al. [69], [70] and Whitmarsh et al. [69], [70] and confirms the evidence of Thiel et al. [71] who demonstrated that activation of the ERK signalling pathway is mainly involved in EGR-1 expression. In contrast, Rolli et al. [72] described a p38 MAPK-dependent induction of EGR-1 expression which was not detected in our HEK293 H_1_R cells.

In mH_4_R-transfected cells, histamine induces only a moderate increase in EGR-1 expression which is also dependent on ERK signalling. So far it is not clear why stimulation with histamine leads to a much lower EGR-1 expression in mH_4_R-transfected cells compared to mH_1_R-transfected cells although in both cell lines ERK is similarly phosphorylated upon HA stimulation. Surprisingly, in mH_4_R-transfected cells, p38 MAPK activity inhibited EGR-1 expression. This was unexpected, since it was not detected in HEK293 H_1_R cells, although in these cells HA induces p38 phosphorylation as well, even stronger as compared to HEK293 mH_4_R cells. In HEK293 mH_1_R cells the p38 MAPK-mediated inhibition of EGR-1 expression may be abolished by the high concentration of cAMP, which is not found in HEK293 mH_1_R cells. Moreover, our finding is also in contrast to the observation by Rolli et al. [72], who demonstrated the induction of EGR-1 expression by p38 MAPK, however using a different stimulus. Consequently, it can be speculated that upon HA-stimulation a mH_4_R-specific pathway is triggered which, via the activity of p38 MAPK, inhibits EGR-1 expression. Thus, in HEK293 mH_1_R cells, which lack this pathway, the p38 MAPK activity lacks a cognate target to reduce ERK1/2 MAPK-induced EGR-1 expression. However, the nature of this mH_4_R-specific pathway has still to be elucidated.

In conclusion, we have shown that HEK293 cells stably expressing comparable quantities of murine histamine receptors represent a valuable model to analyse and directly and reliably compare mH_1_R and mH_4_R mediated signal transduction. To our best knowledge, this is the first published side by side approach analyzing signalling of the pro-inflammatory mH_1_R and mH_4_R using an identical cellular background and comparable expression levels. Using this system, we provide evidence that both the mH_1_R and the mH_4_R mediate the HA-induced [Ca^2+^]_i_ mobilization, the H_1_R being more potent and more efficient. Since both receptors are expressed at comparable quantities, this is either an intrinsic property of the H_1_R, probably indicating that HA-induced coupling of G_q_ proteins is more effective as compared to that of G_i_-proteins, or an intrinsic property of HEK293 cells, probably indicating that in these cells G_q_ proteins are predominant in comparison to G_i_ proteins. In contrast to the mH_1_R, the mH_4_R indeed, as anticipated from the human ortholog, diminishes AC activity, leading to a reduction of cAMP production. Moreover, we demonstrate that the signalling pathways of these receptors are basically homologous, but specific differences, such as at the functional activity of p38 MAPK, which we have newly defined to be involved in mH_4_R signalling, were detected. It will be important to study signal transduction of hH_1_R and hH_4_R according to the same principles as shown here. Comparing signal transduction between human and murine receptor orthologs is essential for proper interpretation of studies in mouse disease models and their translation to human pathology.
